# A highly decoupled and compact co-circularly polarized MIMO filtering antenna array system for vehicular communications

**DOI:** 10.1038/s41598-025-28992-6

**Published:** 2025-11-27

**Authors:** Amit Kumar, Sandeep Rana, Gunjan Srivastava, Akhilesh Mohan, Sachin Kumar, Om Prakash Kumar

**Affiliations:** 1 Department of Electronics and Communication Engineering, Indian Institute of Technology Roorkee, Roorkee, 247667 India; 2 Department of Electronics and Communication Engineering, Graphic Era (Deemed to be) University, Dehradun, 248002 India; 3 Department of Electronics and Communication Engineering, Galgotias College of Engineering and Technology, Greater Noida, 201310 India; 4 Department of Electronics and Communication Engineering, Manipal Institute of Technology, Manipal Academy of Higher Education, Manipal, 576104 India

**Keywords:** MIMO antenna array, Co-circular polarization, Vehicular communications, SDG 9 (Industry, Innovation, and Infrastructure), SDG 11 (Sustainable Cities and Communities), SDG 13 (Climate Action), Optics and photonics, Applied optics

## Abstract

This paper presents a compact, single-layer, and highly decoupled four-element multiple-input-multiple-output (MIMO) filtering antenna array system designed for vehicular communications. A novel sequential phase feed network is developed by replacing the *λ*/4 transformers transmission lines with three-pole hairpin resonator-based band pass filters. The resonators provide filtering characteristics for a 2 × 2 configurations of sequentially rotated corner truncated rectangular patch antennas. The elements of the MIMO filtering antenna array system achieve out-of-band frequency suppression levels exceeding 24 dB in the lower stop band and 36 dB in the upper stop band, demonstrating a sharp filtering response. The MIMO filtering array elements exhibit excellent inter-port isolation, exceeding 34 dB across the entire operating bandwidth of 5.4 GHz to 6.3 GHz. High level of isolation is achieved through two main strategies: first, by rotating the antenna arrays by 120° along the central axis to minimize coupling, and second, by designing a cage to confine the electromagnetic fields. The MIMO filtering array elements have a -10 dB impedance bandwidth of 20%, covering the range from 5.3 GHz to 6.5 GHz, and a 3-dB axial ratio bandwidth of 15.4%, from 5.4 GHz to 6.3 GHz. The elements of the fabricated prototype exhibit a peak realized gain of 11.3 dBic, with an average realized gain of 10.5 dBic. The proposed MIMO filtering antenna array system has the potential to provide the scalability and adaptability needed to support emerging technologies and standards in vehicular communication.This work aligns with the UN Sustainable Development Goals, particularly SDG 9, SDG 11, and SDG 13, by enabling sustainable and intelligent vehicular communications.

## Introduction

Effective communication between the vehicles is crucial for the development of intelligent transportation systems (ITS)^1^. Allowing vehicles to exchange the real-time information can significantly improve the road safety, enhance traffic management, and support advanced features like autonomous driving. One substantial opportunity involves creating a unified antenna system that can serve the multiple vehicular applications. This approach reduces the number of antennas deployed on the vehicles, simplifies the design, lowers the cost, and minimizes the aerodynamic drag.

The 5.4–6.3 GHz frequency band is particularly important for vehicular communications. The sub-band from 5.4–5.7 GHz supports vehicular connectivity through Wi-Fi 5 (IEEE 802.11ac) and Wi-Fi 6 (IEEE 802.11ax), facilitating in-vehicle Wi-Fi, infotainment systems, and fleet management. Additionally, the 5.85–5.925 GHz band has been officially allocated by the Federal Communications Commission (FCC) in the United States and European Telecommunications Standards Institute (ETSI) in Europe for ITS^2,3^, supporting dedicated short-range communications (DSRC) (IEEE 802.11p/802.11bd) and cellular vehicle-to-everything (C-V2X), thereby establishing it as the global vehicular safety spectrum. Moreover, 5.925–6.3 GHz range overlaps with Wi-Fi 6E (IEEE 802.11ax extension), supports next-generation connectivity, enabling seamless, high-speed communication in vehicles and smart transportation hubs. Leveraging the entire 5.4–6.3 GHz band enables the development of a more connected and efficient transportation ecosystem.

To effectively utilize this spectrum, antenna systems must ensure high reliability, large throughput, and robust performance in multipath-rich vehicular environments. In practical scenarios such as dense urban traffic, high-speed highways, and complex road junctions, wireless links experience severe fading, shadowing, and frequent polarization mismatch. Meeting these stringent requirements demands advanced antenna technologies capable of maintaining stable links despite rapid channel variations. The 5G Automotive Association (5GAA) has therefore emphasized that future V2X communication will rely heavily on multi-antenna (multiple-input-multiple-output (MIMO)) systems, which exploit spatial diversity to enhance link robustness, increase capacity, and reduce latency^4^. This system-level requirement sets the context for evaluating antenna designs, for MIMO configurations.

Several linearly polarized (LP) antennas have been reported for vehicular communication in this range. A harmonic-suppressed 4-port LP-MIMO antenna for DSRC applications demonstrates multi-antenna capability^5^. Similarly, a self-decoupled patch system operating in the 5.82–5.98 GHz range achieves excellent isolation (≈78 dB) with strong LP-MIMO performance^6^. In addition, a wideband LP antenna spanning 4–7 GHz confirmed on-vehicle feasibility with a peak gain of 6.23 dBi, though its performance is not analyzed in a multiport environment, limiting its relevance for MIMO-based vehicular systems^7^.

While these works confirm the feasibility of LP antennas for vehicular applications, they also highlight a key limitation: polarization mismatch under dynamic vehicular conditions. To overcome this issue, circular polarization (CP) is explored as a means of mitigating polarization mismatch and enhancing robustness in complex propagation environments, making it particularly attractive for vehicular channels. A polarization-reconfigurable design demonstrated left-hand circular polarization/right-hand circular polarization (LHCP/RHCP) operation across 5.4–6.4 GHz with a peak gain of 6.13 dBic, but it is not validated under multiport conditions^8^. Similarly, a commercially available product, Taoglas SDCP.5900.12.4.A.40 provides RHCP operation for DSRC/C-V2X integration but is also a single-port design without MIMO capability^9^. Such solutions confirm the advantage of CP but do not meet the multi-antenna requirements highlighted by 5GAA. In this direction, a four-port CP-MIMO patch antenna at 5.9 GHz is demonstrated in^10^, validating the feasibility of CP-MIMO links for vehicular applications. However, the design is restricted to a narrow axial ratio bandwidth of 5.82–5.94 GHz, limiting its ability to support the broad range of vehicular application. This limitation underscores the need for wideband CP-MIMO antenna systems capable of supporting multiple vehicular applications. Furthermore, in practical deployment scenarios, the antenna systems must sustain reliable links in multipath-rich environments while handling strong inter-antenna coupling in compact vehicular platforms. This demands high inter-port isolation to preserve MIMO performance and reduce channel correlation. At the same time, long-range V2X links and data-intensive services require high gain to maintain signal quality under interference and fading. To simultaneously address these requirements, the antenna system should integrate polarization robustness, spatial diversity, high inter-port isolation to preserve MIMO diversity performance, and high gain within a compact wideband structure.

As vehicular platforms demand greater RF front-end compactness and integration, antenna systems are increasingly required to accommodate additional functions within a single structure. Among these, filtering has emerged as a key design direction. Incorporating filtering at the antenna level suppresses harmonics and out-of-band signals, reducing dependence on external RF filters and simplifying the overall system architecture. This is especially important in vehicular communication, where multiple wireless standards must operate simultaneously within confined environments. By embedding filtering characteristics, antennas can improve spectral efficiency, mitigate interference between co-located systems, and support compact, reliable, and scalable front-end implementations for ITS applications. In the microstrip antenna technology, three methods exist for integrating filtering functions into antennas^11^. The first method involves adding filtering circuits directly to the antenna, which enhances the antenna’s filtering capabilities but also increases its size^12^. The second method utilizes techniques such as slot loading and shorting-pin loading, which modify the current path of the radiator to create a radiation null, thus eliminating the need for additional filtering circuits^13^. In^14^, the antenna design incorporates a square feeding loop and four L-shaped patches to achieve CP radiation. Band-pass filtering is implemented using four stubs, four shorting pins, and two U-shaped slots. Although this design is single-layered and low-profile, it only achieves a -10 dB impedance bandwidth of 4.5%. This limitation restricts its applicability in systems that require wider bandwidths. However, achieving high levels of suppression with these methods can be challenging. The third method is based on filtering network theory. A two-element circularly polarized filtering patch array based on filtering network theory is discussed in^11^. In this design, the patch antenna is excited using two parallel half-wavelength strips, which are fed by a fork-shaped feedline. This configuration enables multipath coupling between the strips and the patch. While the design achieves two radiation nulls and offers good suppression levels, it is multi-layered and bulky. Antennas designed with substrate integrated waveguide (SIW) technology can also effectively achieve cross-coupling or mixed-coupling, enables to create the radiation nulls^15^. A common approach in this category involves multilayered 3D configurations, where resonators are stacked vertically. In^16^, the antennas utilize a dual-mode SIW cavity (TE_102_/TE_201_) that acts as both resonators and an excitation source. Sparse vias around the cavity enable controlled magnetic leakage to excite four orthogonal quarter-wavelength patches, which receive equal amplitude fields with a 90° phase shift for circular polarization. This arrangement combines cavity resonance with radiating patches to create a filtering effect, while asymmetrical shorting pins and a folded structure improve impedance matching, axial ratio, and directivity. However, the axial ratio bandwidth is relatively narrow at 3.9%, potentially limiting its use in wideband systems, and the folded multilayer design may complicate fabrication and alignment. In^17^, the antennas operate by vertically integrating a single-fed circularly polarized patch antenna with two high-Q SIW cavity resonators to form a third-order Chebyshev filtering response. While these multilayered designs can improve performance, they significantly increase the size and complexity of fabrication, making integration with other RF components more difficult. Consequently, filtering antennas often face challenges balancing a compact size with a simple single-layer structure while providing effective out-of-band suppression. This challenge also affects the adaptability of filtering antenna designs in higher-order MIMO systems. Furthermore, incorporating the filtering characteristics into circularly polarized antenna arrays for MIMO technology complicates the design further. When MIMO antennas are placed closely together, inter-port isolation decreases, adding another layer of complexity. A hybrid isolation enhancement technique for a two-port, dual-circularly polarized antenna array for MIMO systems is discussed in^18^. The design achieves a minimum isolation of 24 dB across the operating band of 4.768–4.92 GHz by utilizing defected ground structure (DGS)-based slots and an epsilon-and-mu-near-zero metasurface superstrate. However, the use of DGS can lead to back radiation leakage, while the superstrate increases the overall height and fabrication complexity. Additionally, misalignment during assembly can negatively affect performance. A two-port LP filtering antenna array with hairpin resonators is designed for vehicular applications in^19^. In this system, the transmitting (*Tx*) and receiving (*Rx*) antennas operate within different frequency ranges: 3.41–3.76 GHz for *Tx* and 5.65–6.1 GHz for *Rx*, or vice versa. This frequency separation reduces sensitivity to signals outside their designated ranges to achieve a minimum isolation of 30 dB, though it is a spectrally inefficient technique for minimizing inter-port coupling. Furthermore, the system incorporates a multi-layer design, which increases fabrication complexity and necessitates precise alignment of the layers to ensure optimal performance. Therefore, designing a wideband highly isolated MIMO antenna system that features circularly polarized arrays with filtering characteristics, all on a single layer and in a compact size, poses a significant challenge.

To overcome these challenges, this article presents a compact, single-layer, and highly decoupled four-element co-circularly polarized MIMO filtering antenna array system designed for vehicular communication operating in 5.4–6.3 GHz frequency range. A novel sequential phase feed network is developed by replacing the *λ*/4 transformers with three-element hairpin resonators-based filtering structures. The resonators provide filtering characteristics for a 2 × 2 configuration of sequentially rotated truncated corner rectangular patches. Subsequently, a four-element MIMO filtering antenna array system is designed, demonstrating high inter-port isolations. This high isolation is achieved through two key strategies: (i) rotating the antenna arrays by 120° to minimize couplings, and (ii) designing a cage to confine the electromagnetic fields. Simulated and measured results are in close agreement, validating the effectiveness of the proposed structure. The proposed antenna system integrates four essential features within a compact configuration: polarization robustness through circular polarization, spatial diversity and link reliability enabled by MIMO technology, intrinsic out-of-band suppression through the filtering feed network, and wide operating band with high gain across the ITS and Wi-Fi spectrum of interest. Together, these characteristics ensure reliable operation across diverse vehicular scenarios, while offering the scalability and adaptability required to support emerging technologies and future standards in intelligent transportation systems.

## Antenna configuration

**Fig. 1:**
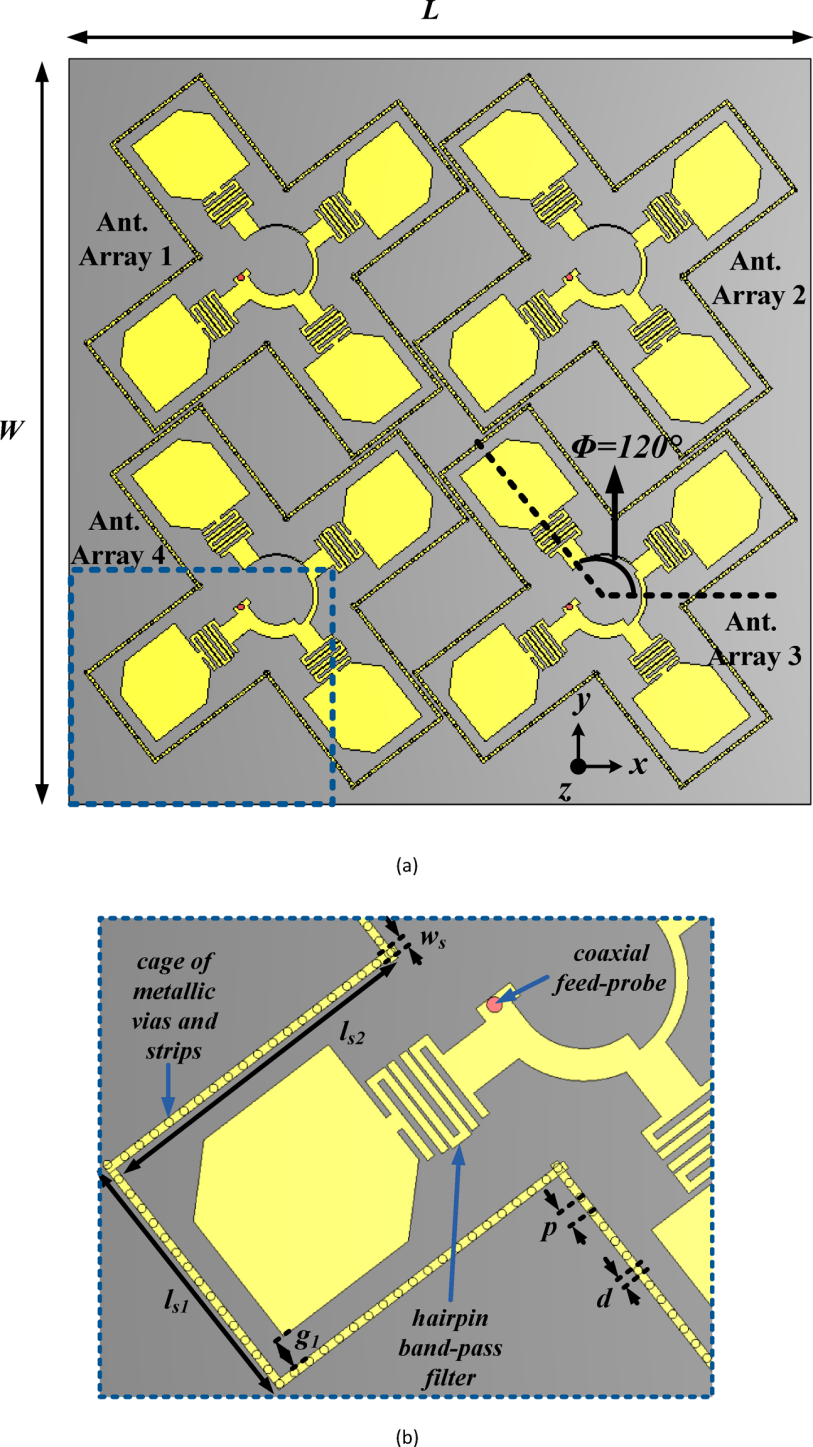
Proposed MIMO filtering antenna array system, and (b) zoomed view of the section highlighted in blue (All dimensions are in mm): *L* = 150, *W* = 150, *l*_*s*1_ = 22.9, *l*_*s*2_ = 29, g_1_ = 2.4, *w*_*s*_ = 0.7, *p* = 1.51, and *d* = 0.7.

Figure 1 illustrates the proposed four-element, co-circularly polarized MIMO filtering antenna array system for vehicular communications. The MIMO antenna system is designed on Rogers RT/Duroid 5880 substrate (*ε*_*r*_ = 2.2, thickness = 1.575 mm, and tan *δ* = 0.0009) with a ground plane of size *L* × *W* (150 mm × 150 mm). Due to lower relative permittivity, low loss tangent, minimal moisture absorption, and wide range of thermal stability, the RT/Duroid 5880 substrate is chosen. It consists of four coaxial-fed antenna arrays, each comprising a 2 × 2 arrangement of sequentially rotated, corner-truncated rectangular patch antennas. Each array has a feed network incorporating four 3-pole hairpin band pass filters, providing the necessary filtering characteristics. The four elements of the MIMO antenna systems are placed closely with a rotation of *ϕ* = 120° along the central axis to minimize inter-port couplings. Moreover, a series of shorted metallic vias and strips are utilized to construct a cage that confines the electromagnetic fields within it, which further reduces the inter-port coupling.

### Filtering antenna unit element array

This section describes the configuration and operating principle of the unit element array in the proposed co-circularly polarized MIMO filtering antenna array system. It can be divided into two stages. Initially, a 2 × 2 array of sequentially rotated circularly polarized antennas is developed in Stage A. Subsequently, hairpin band pass filters are integrated into the feed network, to obtain the filtering characteristics in Stage B.

#### Stage A

**Fig. 2:**
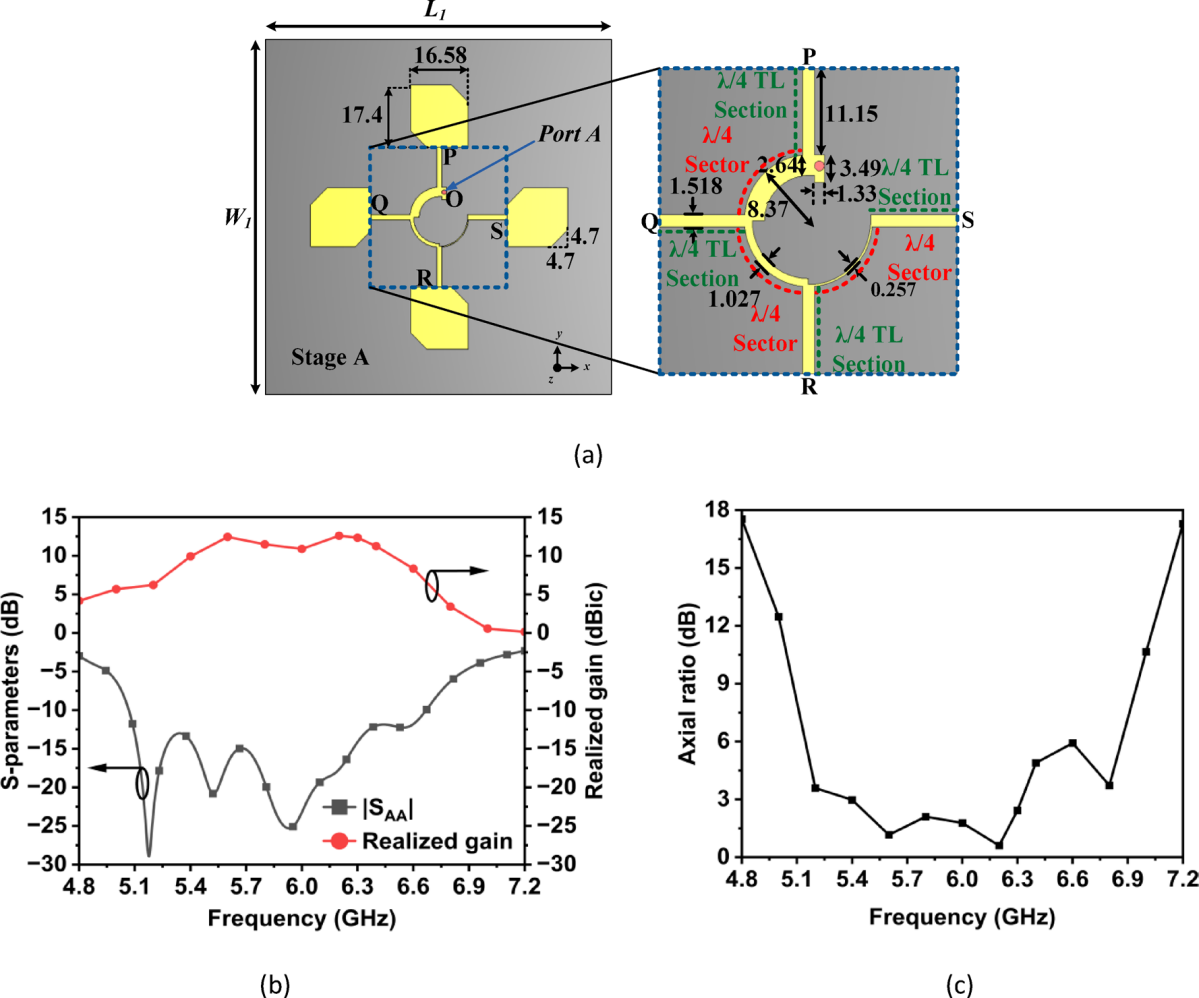
(**a**) Antenna structure of Stage A, (**b**) |S_AA_|, realized gain, and (**c**) axial ratio (All dimensions are in mm): *L*_1_ = 100 and *W*_1_ = 100.

Figure 2a illustrates the schematic layout of a 2 × 2 sequentially rotated right-handed circularly polarized antenna array. The proposed array consists of four corner truncated rectangular patches, each measuring 17.4 mm by 16.58 mm, with a corner truncations of 4.7 mm. The patches are arranged orthogonally and are fed through a sequential power divider. At the feed point *O*, as shown in Fig. 2a, the input impedance of the power divider is 50 Ω, while the four output ports [labeled as *P*, *Q*, *R*, and *S* in Fig. 2a] have an impedance of 115 Ω. The *λ*/4 sectors provide a phase shift of 90°, resulting in the following phase relationships: 90° at point *Q*, 180° at point *R*, and 270° at point *S*, all referenced to point *P*. Four *λ*/4 transmission line sections are utilized to transform an impedance of 82 Ω at the *λ*/4 sectors to 115 Ω at the input of patches. This transformation is essential for matching the impedance between the sectors and the truncated corner rectangular patch antennas. The input impedance of the truncated corner rectangular patches is approximately 115 Ω, which facilitates proper impedance matching with the outputs of the sequential power divider. The primary function of the sequential power divider is to evenly distribute power among the four radiating patches while providing a 90° progressive phase shift in the array and performing the necessary impedance transformation from 50 Ω to 115 Ω. The input reflection coefficient of the designed antenna unit element array is shown in Fig. 2(b). This array exhibits an excellent impedance matching within the operating band of 5.4 to 6.3 GHz, with |S_AA_| ≤ -13 dB. The average realized gain exceeds 11.3 dBic, with a peak value of 12.6 dBic, while the axial ratio remains below 3 dB across the entire operating frequency range, as illustrated in Fig. 2(c).

#### Stage B

**Fig. 3:**
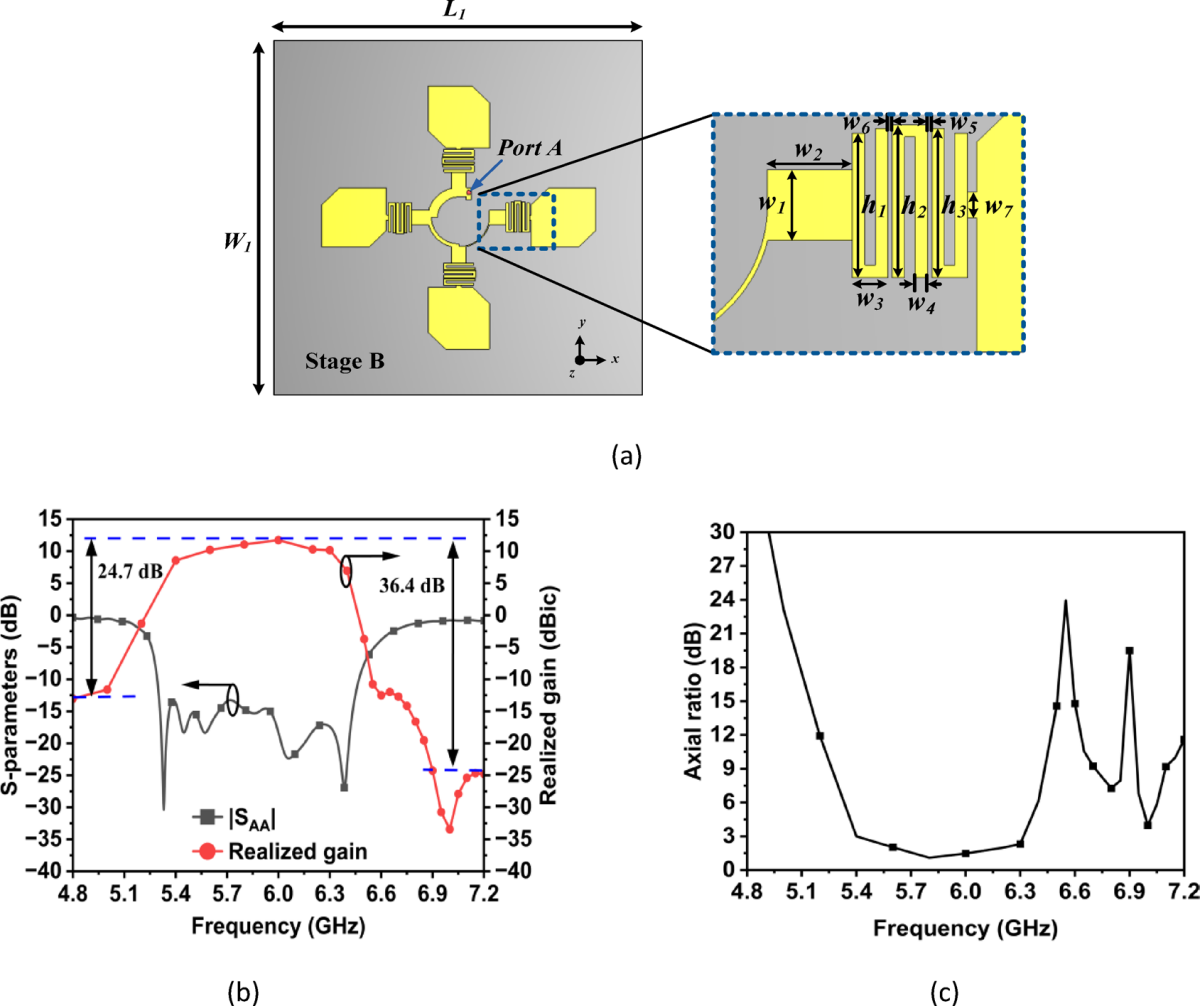
(**a**) Antenna structure of Stage B and zoomed view of band pass filter, (**b**) |S_AA_|, realized gain and (**c**) axial ratio of Stage B (All dimensions are in mm): *L*_1_ = 100, *W*_1_ = 100, *w*_1_ = 4, *w*_2_ = 4.61,* w*_3_ = 1.96, *w*_4_ = 0.69, *w*_5_ = 0.21, *w*_6_ = 0.2, *w*_7_ = 1.47, *h*_1_ = 8.16, *h*_2_ = 8.64, *h*_3_ = 8.43.

**Fig. 4:**
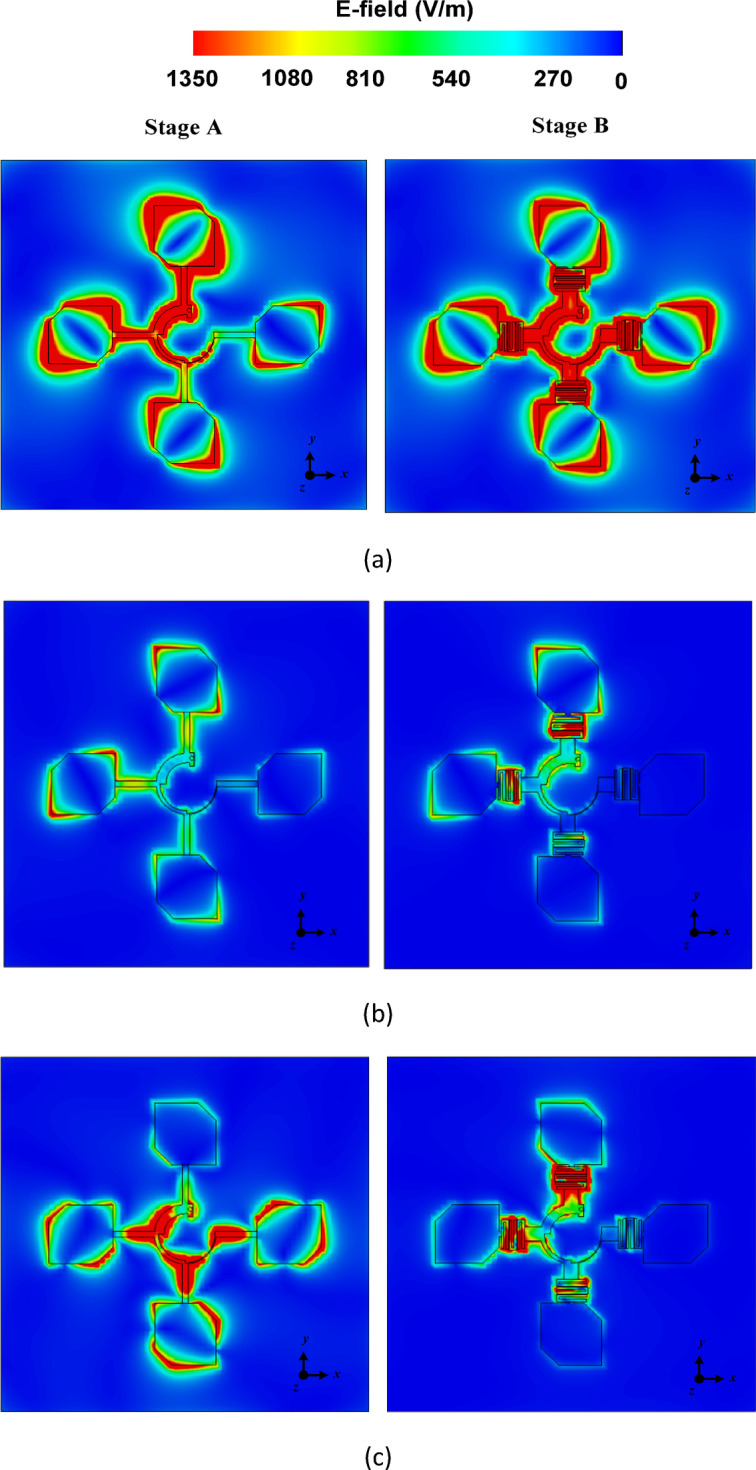
E-field distributions at (**a**) 5.9 GHz (pass band), (**b**) 4.8 GHz (lower stop band), and (**c**) 6.8 GHz (upper stop band).

In Stage B, as shown in Fig. 3, the *λ*/4 transmission line sections are replaced with a 3-pole hairpin band pass filters (BPFs). The length of the hairpin resonators can be calculated using the relation in the reference^20^_._ Furthermore, the widths of the input and output transmission lines (TLs) are optimized to ensure compatibility between the input and output impedances of both the sectors and the patch antennas. The input reflection coefficient, realized gain and axial ratio for the filtering antenna unit element array (Stage B) are illustrated in Fig. 3. Within the operating frequency band of 5.4 to 6.3 GHz, |S_AA_| ≤ -13 dB. The realized gain is better than 8.5 dBic, with a peak value of 11.74 dBic at 6 GHz. Furthermore, the axial ratio also remains below 3 dB throughout the operating frequency range. To understand the role of hairpin BPFs, the E-field distributions for both the stages, are plotted at three different frequencies i.e. in the pass band (5.9 GHz), in the lower stop band (4.8 GHz) and in the upper stop band (6.8 GHz). As illustrated in Fig. 4a, Stage A and Stage B exhibit identical E-field distributions in the pass band at 5.9 GHz, indicating that the filter effectively allows this frequency to pass through it. In contrast, in the lower and upper stop bands at 4.8 GHz and 6.8 GHz, respectively, the E-fields are mainly concentrated around the regions of the hairpin resonators, as shown in Fig. 4b and c. This observation confirms that the filter successfully attenuates the frequencies that lies outside the pass band. The designed filtering unit element antenna array of Stage B achieves out-of-band suppression levels exceeding 24 dB at the lower edge (4.8 GHz) and 36 dB at the upper edge (6.8 GHz) of the pass band which demonstrates its sharp filtering characteristics.

### Equivalent circuit model of the filtering antenna unit element array

In this section, the equivalent circuit model of the filtering antenna unit element array is presented. Initially, the corner truncated rectangular patch is modeled independently along with its feedline and the feed-patch discontinuity. Subsequently, the sequential feed network, hairpin band pass filters and corner truncated rectangular patch are modeled together to obtain the complete equivalent circuit representation of the filtering antenna element array.

### Corner truncated rectangular patch antenna along with its feedline

**Fig. 5:**
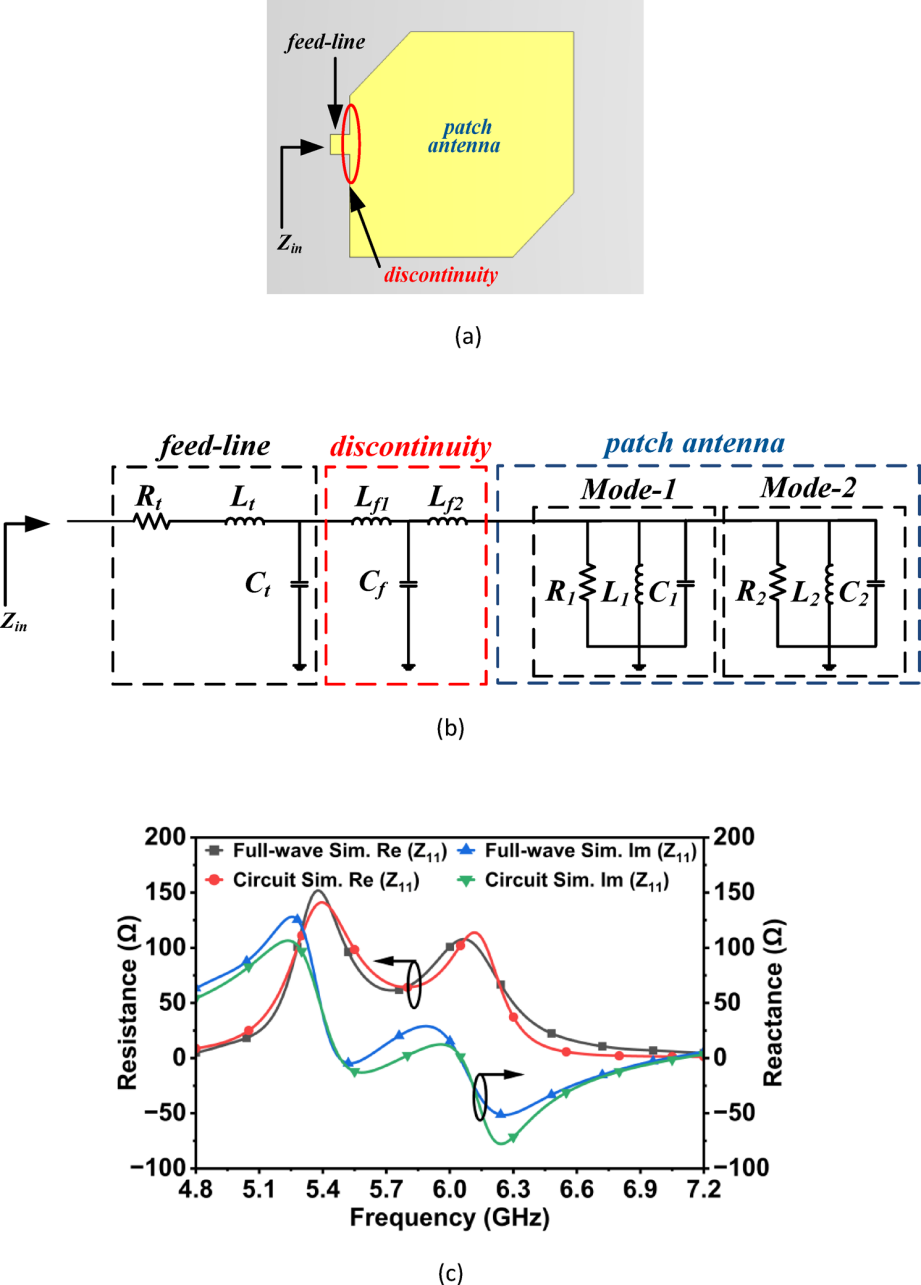
(**a**) Antenna section, (**b**) RLC equivalent, and (**c**) simulated impedance (*R*_*t*_ = 0.999997 Ω, *L*_*t*_ = 0.999975 nH, *C*_*t*_ = 0.999983 pF, *L*_*f*1_ = 1.45791 nH, *C*_*f*_ = 1.51364 pF, *L*_*f*2_ = 1.63041 nH, *R*_1_ = 129.847 Ω, *L*_1_ = 2.93729 nH, and *C*_1_ = 2.7065 pF, *R*_2_ = 119.996 Ω, *L*_2_ = 0.264212 nH, and *C*_2_ = 1.00389 pF).

The antenna section modeling comprises three components: the feed line, the feed-patch discontinuity, and the corner-truncated rectangular patch radiator as shown in Fig. 5a. The input feed line section (*w*_7_ = 1.47 mm, *l*_7_ = 0.5 mm, and *h* = 1.575 mm) is modeled as a RLC network. The network includes series resistance, inductance, and shunt capacitance to account for losses and stored energy. The input feed line’s parameters (*L*_*t*_, *C*_*t*_, and *R*_*t*_) are calculated from transmission line theory^21^.

The discontinuity between the feed line and the wide patch is modeled as a T-network, consisting of a shunt capacitance and two series inductances. The T-network represents the fringing fields and current spreading at the feed transition. The discontinuity parameters (*L*_*f*1_,* C*_*f*_, and* L*_*f*2_) are calculated using Eqs. (1)–(6)^20^.

1 $$C_{f} = 0.00137h \frac{{\sqrt {\varepsilon_{eff1} } }}{{Z_{c1} }}\left( {1 - \frac{{w_{2} }}{{w_{1} }}} \right)\left( {\frac{{\varepsilon_{eff1} + 0.3}}{{\varepsilon_{eff1} - 0.258}}} \right)\left( {\frac{{\frac{{w_{1} }}{h} + 0.264}}{{\frac{{w_{1} }}{h} + 0.8}}} \right){\text{pF}}$$

2 $$L_{f1} = \frac{{L_{w1} }}{{L_{w1} + L_{w2} }}L_{d}$$

3 $$L_{f2} = \frac{{L_{w2} }}{{L_{w1} + L_{w2} }}L_{d}$$

4 $$L_{w1} = \frac{{Z_{c1} \sqrt {\varepsilon_{eff1} } }}{c}$$

5 $$L_{w2} = \frac{{Z_{c2} \sqrt {\varepsilon_{eff2} } }}{c}$$

6 $$L_{d} = 0.000987h\left( {1 - \frac{{Z_{c1} }}{{Z_{c2} }}\sqrt {\frac{{\varepsilon_{eff1} }}{{\varepsilon_{eff2} }}} } \right)^{2} {\text{nH}}$$

where ${Z}_{c1}$ and ${\varepsilon }_{eff1}$ are impedance and effective permittivity of wide output line (patch antenna of width 17.4 mm) and ${Z}_{c2}$ and ${\varepsilon }_{eff2}$ are impedance and effective permittivity of narrow input line (input feedline *w*_7_ = 1.47 mm). The truncated patch excites two orthogonal resonances due to the corner cut, each represented by a parallel RLC branch with impedance given by Eq. (7).

7 $$Z_{{RLC_{i} }} \left( j \right) = \frac{1}{{\frac{1}{{R_{i} }} + j\left( {C_{i} - \frac{1}{{L_{i} }}} \right)}}\;{\text{where}}\;i = 1 , 2$$

Figure 5c represents the full-wave and circuit simulated *Z*_11_-parameters of the corner truncated rectangular patch along with its feedline. As illustrated in the figure, the simulated input impedances confirm the excitation of two orthogonal resonant modes: one at 5.48 GHz and another at 6.05 GHz. These modes correspond to the perturbed TM_10_ and TM_01_ modes of the rectangular patch, which are split due to corner truncation. Therefore, the RLC equivalent circuit of the antenna section elucidates the resonance mechanism and provides a strong foundation for achieving wideband circularly polarized radiation over the 5.4–6.3 GHz range through the sequential feeding technique.

### Sequential feed network, hairpin band pass filters and corner truncated rectangular patch antenna

**Fig. 6:**
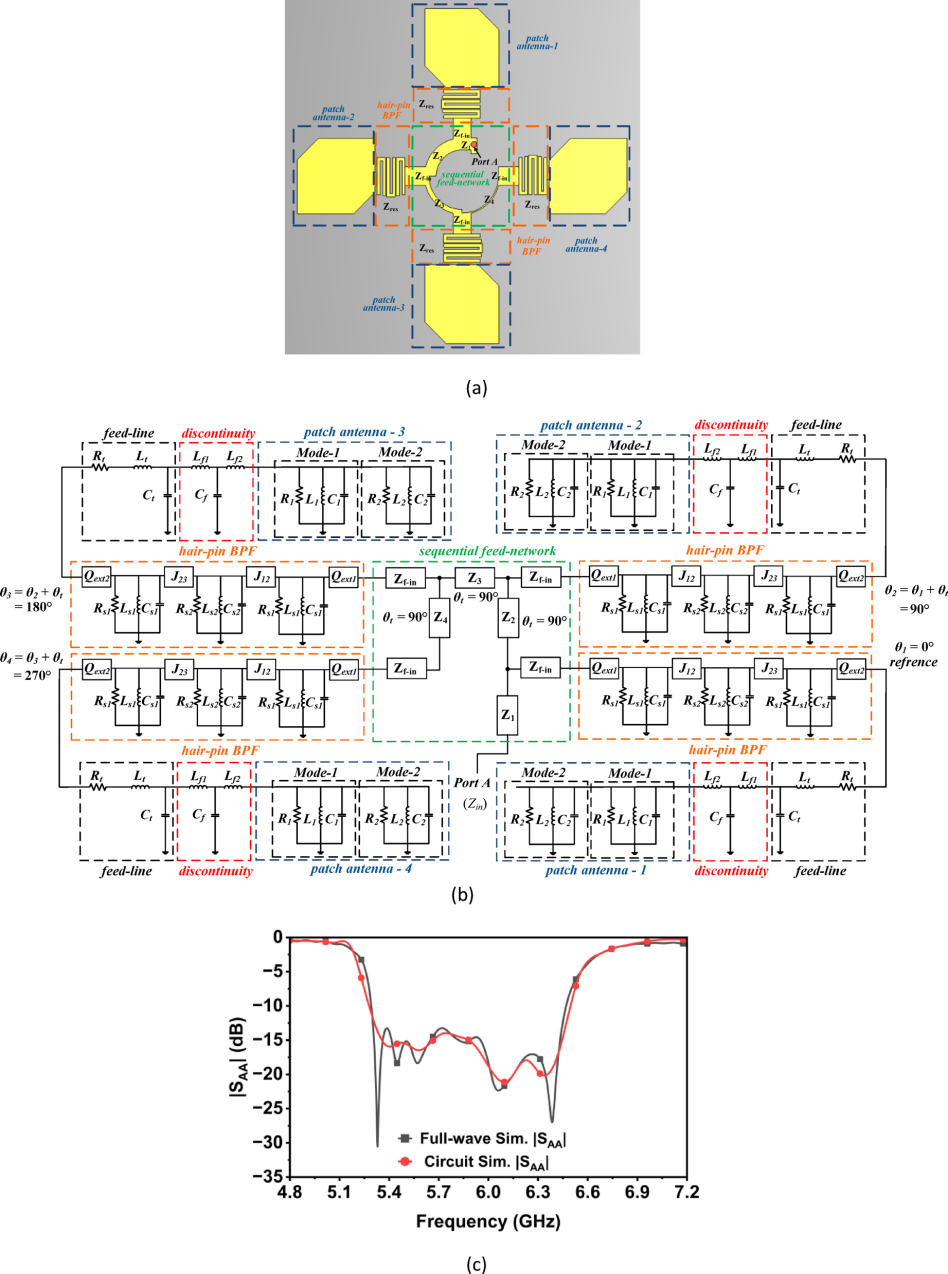
(**a**) Filtering antenna unit element array, (**b**) its equivalent circuit diagram, (**c**) |S_AA_| at Port A (*R*_*s*1_ = 0.6616 kΩ, *L*_*s*1_ = 3.6227 nH, *C*_*s*1_ = 0.20431 pF, *R*_*s*2_ = 0.7453 kΩ, *L*_*s*2_ = 4.08 nH, *C*_*s*2_ = 0.1959 pF, *Z*_*res*_ = 133.16 Ω, *Z*_*f-in*_ = 57.08 Ω, *J*_12_ = 0.323665, *J*_23_ = 0.2418812, *Q*_*ext*1_ = 7.1771356, *Q*_*ext*2_ = 4.0083341, *Z*_2_ = 73 Ω, *Z*_3_ = 114 Ω, *Z*_4_ = 180 Ω).

Figure 6b represents the equivalent circuit model of the proposed CP filtering antenna unit element array shown in Fig. 6a. The antenna unit element integrates sequential feed network, hair-pin band pass filters and corner truncated rectangular patches. The sequential feed network can be modeled using impedance-transforming transmission-line sections, whereas the hair-pin band pass filter consists of three coupled RLC tank circuit with admittance inverters and external quality factors. The equivalent circuit model of corner truncated rectangular patch is already presented in the above sub-section. The sequential feed network begins at Port A, corresponding to the 50-Ω SMA input. The first transmission line, *Z*_1_, models the combined effects of the SMA pad and the short launch section that connects the connector to the feed network. *Z*_*f-in*_ denotes the effective impedance presented to the subsequent filtering stage. Equal power division is achieved through a symmetric four-way topology, where the transmission lines are designed with different characteristic impedances. This design ensures that each branch maintains the same amplitude at its output. Phase progression is implemented by transformers *Z*_2_, *Z*_3_, and *Z*_4_, which introduce successive 90° electrical delays at the operating frequency. As a result, the four outputs exhibit sequential phase states of 0°, 90°, 180°, and 270°, all with equal magnitudes. The presented design adheres to the well-established sequential rotation principle widely used in broadband circularly polarized microstrip arrays. The filtering section is implemented by a three-pole hairpin band pass filter integrated between the sequential feed and the truncated patch radiator. Each hairpin is modeled as a parallel RLC tank, with inductance and capacitance calculated from the resonant impedance and design frequency, and resistance accounting for finite conductor and dielectric losses. The two outer resonators share identical dimensions, whereas the central resonator is slightly longer to control the symmetry of the pass band. Inter-resonator coupling is represented by admittance inverters *J*_12_ and *J*_23_, which are implemented in the lumped circuit by equivalent series capacitors. Similarly, the port terminations are captured through the external quality factors *Q*_*ext*1_ and *Q*_*ext*2_, realized by series coupling capacitors to the ports. Using the 0.05-dB ripple Chebyshev prototype coefficients and a fractional bandwidth of 20.1%, the complete set of RLC values, inverter constants, and external quality factors are calculated and implemented in the circuit model^20^. When combined, the sequential feeding network, the three-pole hairpin band pass filters, and the corner-truncated patch radiator form a complete lumped-element model of the filtering antenna unit element array. Circuit simulations of this synthesized network exhibit input-matching and resonance characteristics that are in close agreement with the corresponding full-wave EM results as shown in Fig. 6c, confirming the accuracy of the equivalent representation.

### Proposed four-element MIMO antenna array system

**Fig. 7:**
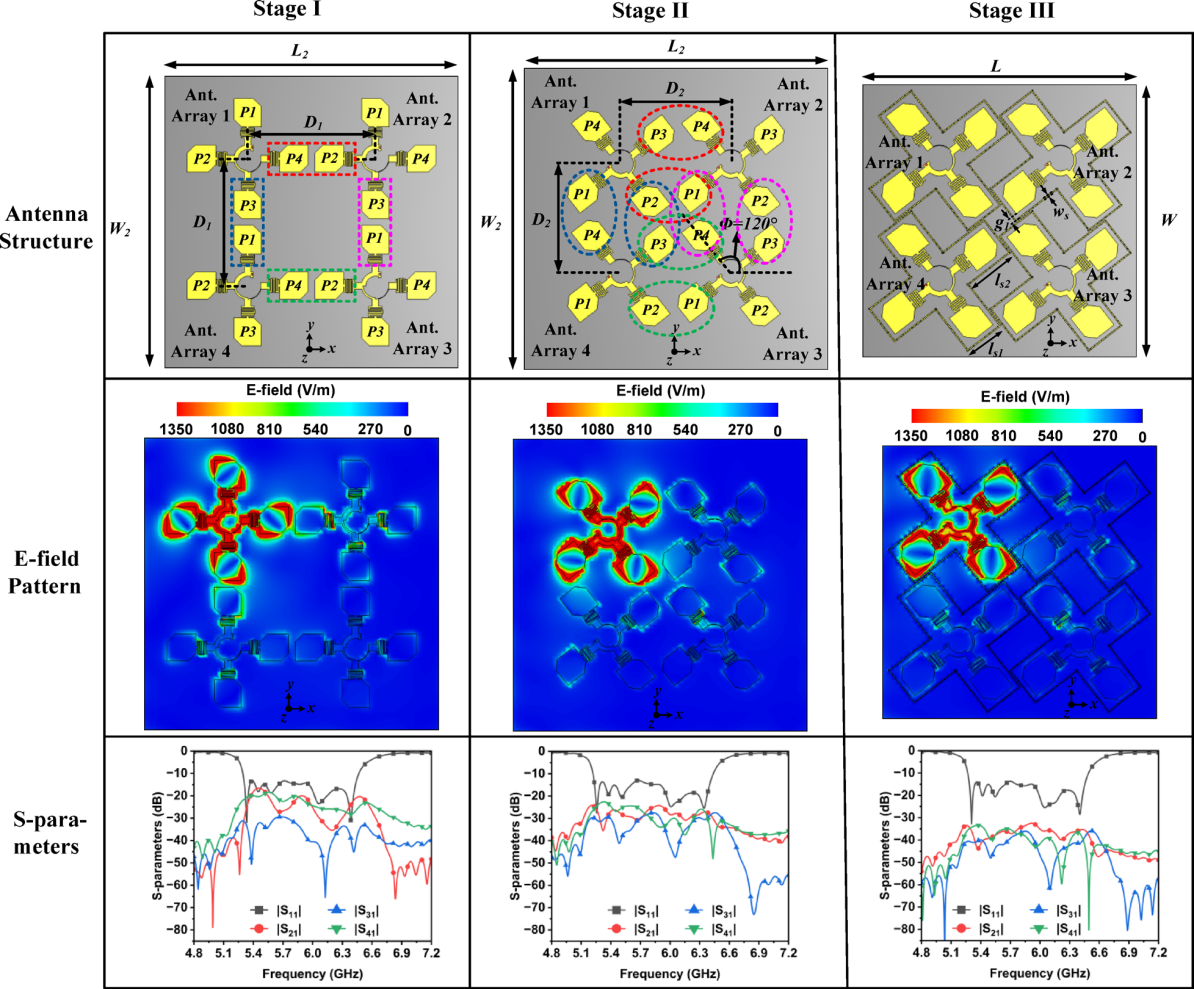
Design stages of the proposed four-element MIMO filtering antenna array system, their E-field distribution at 5.9 GHz and S-parameters (Dimensions in mm): *L* = 150, *W* = 150, *l*_*s*1_ = 22.9, *l*_*s*2_ = 29, g_1_ = 2.4, *w*_*s*_ = 0.7, *D*_1_ = 80, *D*_2_ = 60, *L*_2_ = 180, and *W*_2_ = 180.

The proposed four-element co-circularly polarized MIMO filtering antenna array system is designed in three stages as shown in Fig. 7. Initially, in Stage I, four filtering antenna unit element array (as designed in Stage B) are arranged with a center-to-center spacing, *D*_1_ = 80 mm. In Stage II, the filtering antenna unit element arrays are rotated by 120° along its central axis to reduced mutual couplings. The arrays are moved closer to each other with a center-to-center spacing, *D*_2_ = 60 mm to create a more compact antenna system. Finally, in Stage III, a series of grounded vias and strips are employed to confine the electromagnetic field, further enhancing isolation between the ports.

#### Stage I

In Stage I, four units of the filtering antenna array, developed in Stage B, are arranged with a center-to-center spacing of *D*_1_ = 80 mm. It is observed that several pairs of truncated corner patch antennas from the antenna arrays (AAs) are in close proximity to each other. For example, patch P4 of AA1 is near patch P2 of AA2, and patch P3 of AA1 is close to patch P1 of AA 4, and so on, as illustrated in Fig. 7. The electric fields of all these adjacent patches are aligned in the same plane, facilitating strong near-field interactions. In this configuration, fringing fields, surface waves, and edge diffraction effectively couple energy between the patch antennas, resulting in increased mutual coupling. Inter-port isolation of better than 16 dB is achieved across the entire operating band of 5.4 to 6.3 GHz at this stage.

#### Stage II

As discussed above, the Stage II focuses on creating a more compact antenna system along with the improved inter-port isolations. In this stage, the filtering antenna unit element arrays are rotated 120° along their central axis, and the spacing between them is decreased by 20 mm to obtain a more compact design. This configuration brings two pairs of patches from each antenna array in close proximity with adjacent antenna arrays. For example, between AA1 and AA2, patch antenna pairs P3 and P4, as well as P2 and P1, are positioned close to each other. This pattern is consistent across all pairs of adjacent antenna arrays, as illustrated in Fig. 7. Nevertheless, the two pairs of patch antennas that are in close contact in Stage II are oriented with orthogonal E-planes. This orthogonal configuration minimizing induced currents in adjacent antennas that enhances the inter-port isolation. Additionally, the orthogonal arrangement also reduces direct aperture-to-aperture interactions which effectively suppresses near-field couplings. As a result, inter-port isolations exceeding 25 dB over the entire operating band of 5.4 to 6.3 GHz, at this stage, is obtained.

#### Stage III

Stage III aims to further improve inter-port isolation. In this stage, a series of shorted metallic vias and strips form a cage that confines electromagnetic waves. These vias and connecting strips establish a low-impedance pathway for electromagnetic waves. The vias serve as vertical grounding elements that inhibit wave propagation by providing a low-impedance route to the ground plane, while the strips help distribute the fields across the series of vias. This arrangement results in effective electromagnetic shielding and contributes to broad-spectrum interference suppression. The shorting vias have a diameter of 0.7 mm, with a gap of 1.51 mm between them, yielding a diameter-to-gap ratio of approximately 0.46. As shown in Fig. 7, the designed cage effectively captures the electromagnetic fields within it, significantly reducing coupling between the antenna arrays. At this stage, inter-port isolation of better than 34 dB is achieved.

#### Vehicular integration

**Fig. 8:**
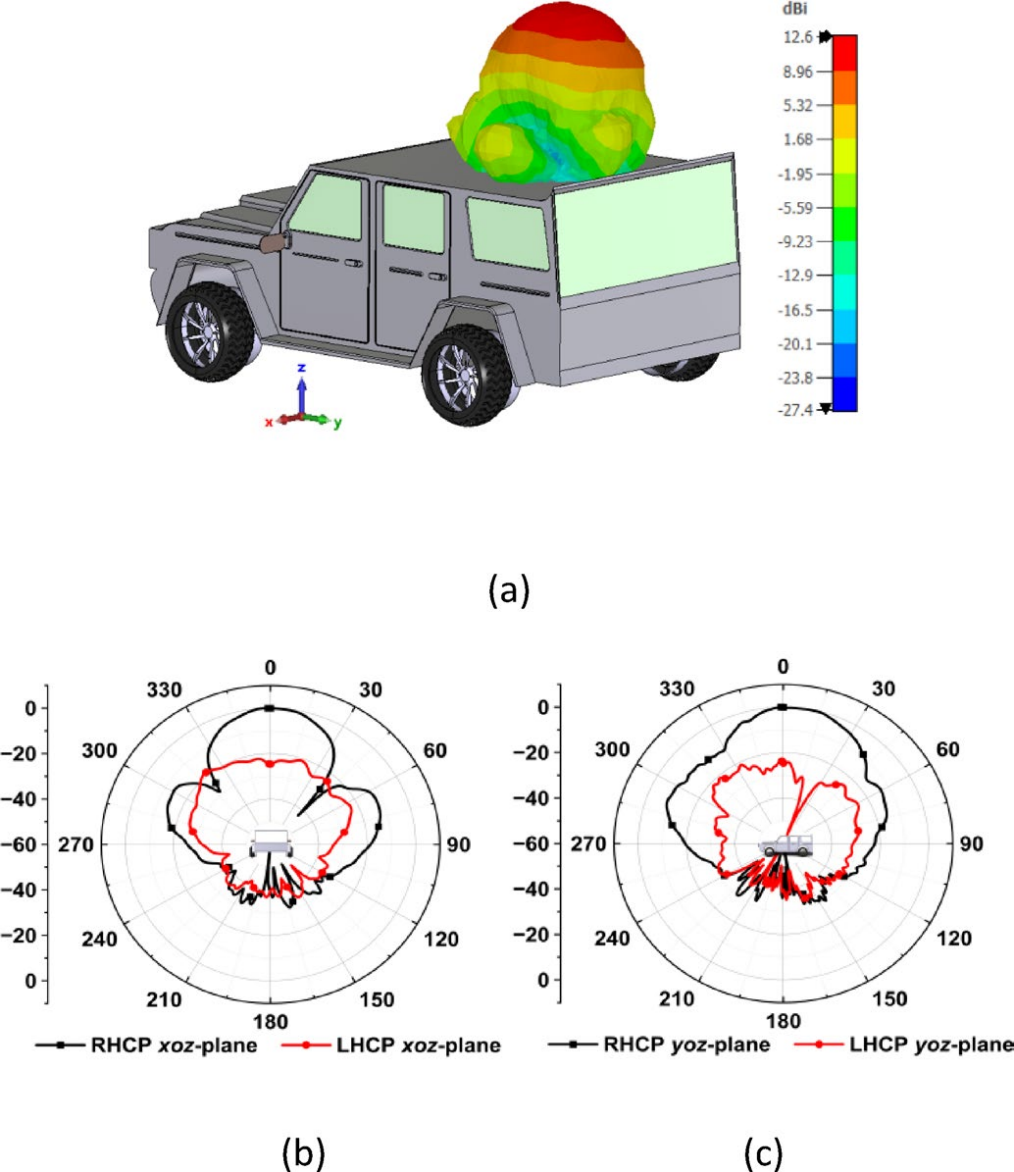
(**a**) 3D realized gain on the roof (horizontal arrangement) of the vehicle at 5.9 GHz, (**b**) 2D normalized radiation pattern in *xoz*-plane, and (c) *yoz*-plane.

**Fig. 9:**
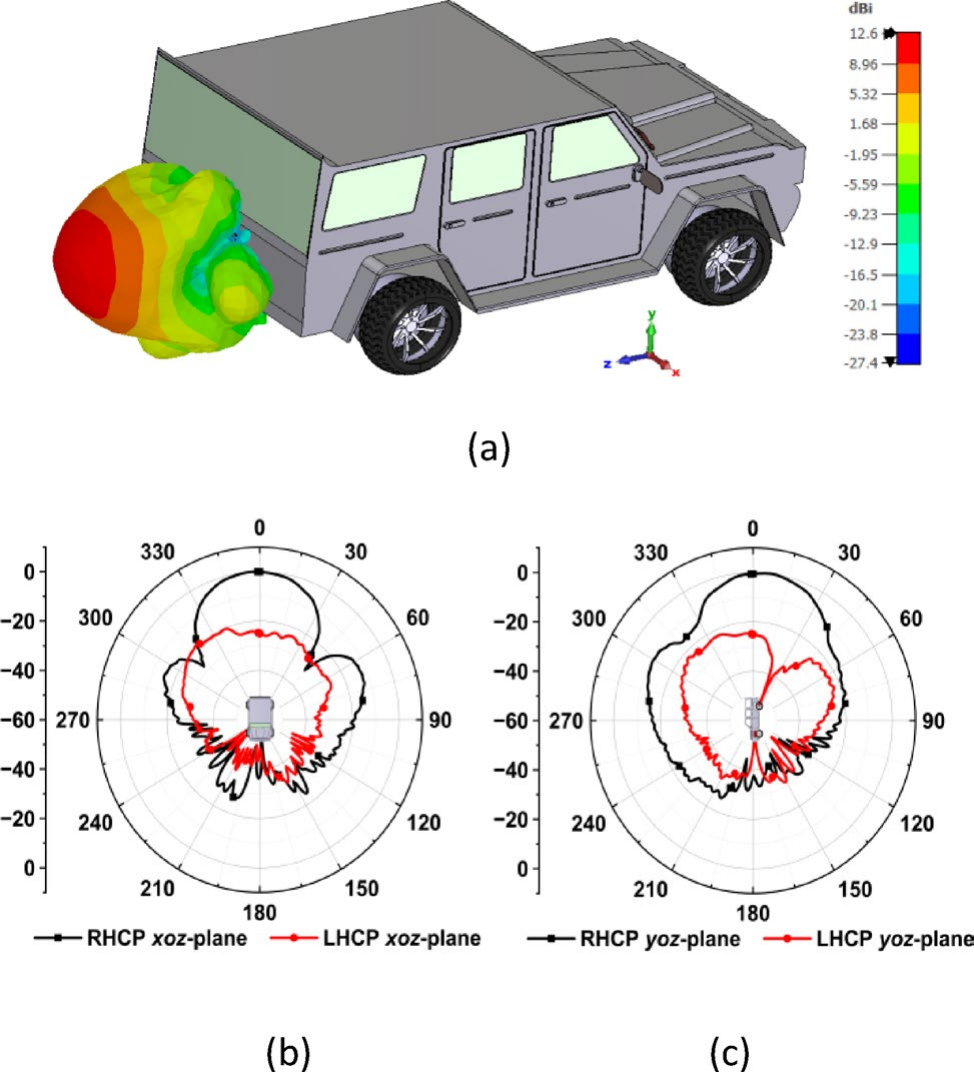
(**a**) 3D realized gain on the rear bumper (vertical arrangement) of the vehicle at 5.9 GHz, (**b**) 2D normalized radiation pattern in *xoz*-plane, and (**c**) *yoz*-plane.

To enhance coverage performance, multiple MIMO antennas are strategically placed on a vehicle, including the roof, side doors, front bumper, and rear bumper. This arrangement creates a distributed MIMO (D-MIMO) setup, which significantly improves signal quality and reduces dead zones through spatial multiplexing and beamforming, making it ideal for reliable vehicular communication. The surface features of the vehicle affect the radiation properties of the antennas. Metallic components can reflect and scatter electromagnetic waves, resulting in variations depending on the antennas’ placement. For brevity, in this study, the radiation characteristics of the proposed MIMO antenna system are analyzed at two specific locations on the vehicle: the roof (with a horizontal arrangement) and the rear bumper (with a vertical arrangement), as shown in Figs. 8 and 9, respectively. This analysis is performed using a CAD model of the vehicle, modeling the body as a perfect electric conductor (PEC) while assigning other components materials based on their specific properties. The proposed MIMO antenna demonstrates a peak realized gain of 11.74 dBic. When the antenna is mounted on the vehicle, the effective ground plane expands due to the surrounding metallic surfaces. This improvement enhances the antenna’s directivity, increasing the realized gain to approximately 12.6 dBic for both roof and rear bumper placements.

In the roof-mounted configuration, the antenna radiates a broadside RHCP beam with a high front-to-back ratio and a relatively smooth radiation envelope. However, moderate ripples can be observed in the side lobes due to edge diffraction. In the rear bumper configuration, the antenna produces a forward-directed RHCP-dominant beam that exhibits comparable gain and angular coverage to the roof-mounted configuration. Nevertheless, the side lobes of the RHCP pattern show slightly stronger ripples than those of the roof-mounted configuration. This effect is due to scattering caused by the edges of the bumper and nearby structures, which have a relatively smaller ground plane extension.

Despite the localized variations, both the configurations maintain RHCP dominance in their main lobes, with cross polarization discrimination (XPD) over 22 dB, indicating stable circular polarization purity. Maintaining this RHCP purity is crucial in vehicular channels, where multipath propagation and scattering can alter the polarization sense. The antenna minimizes polarization mismatch losses and enhances link reliability under dynamic channel conditions by radiating a strong RHCP-dominant direct path. The results demonstrate that integrating the antenna with the vehicle increases its peak gain compared to the free-space scenario while preserving the intended polarization performance. This highlights the robustness of the proposed antenna system in a vehicular environment.

## Results and discussion

**Fig. 10:**
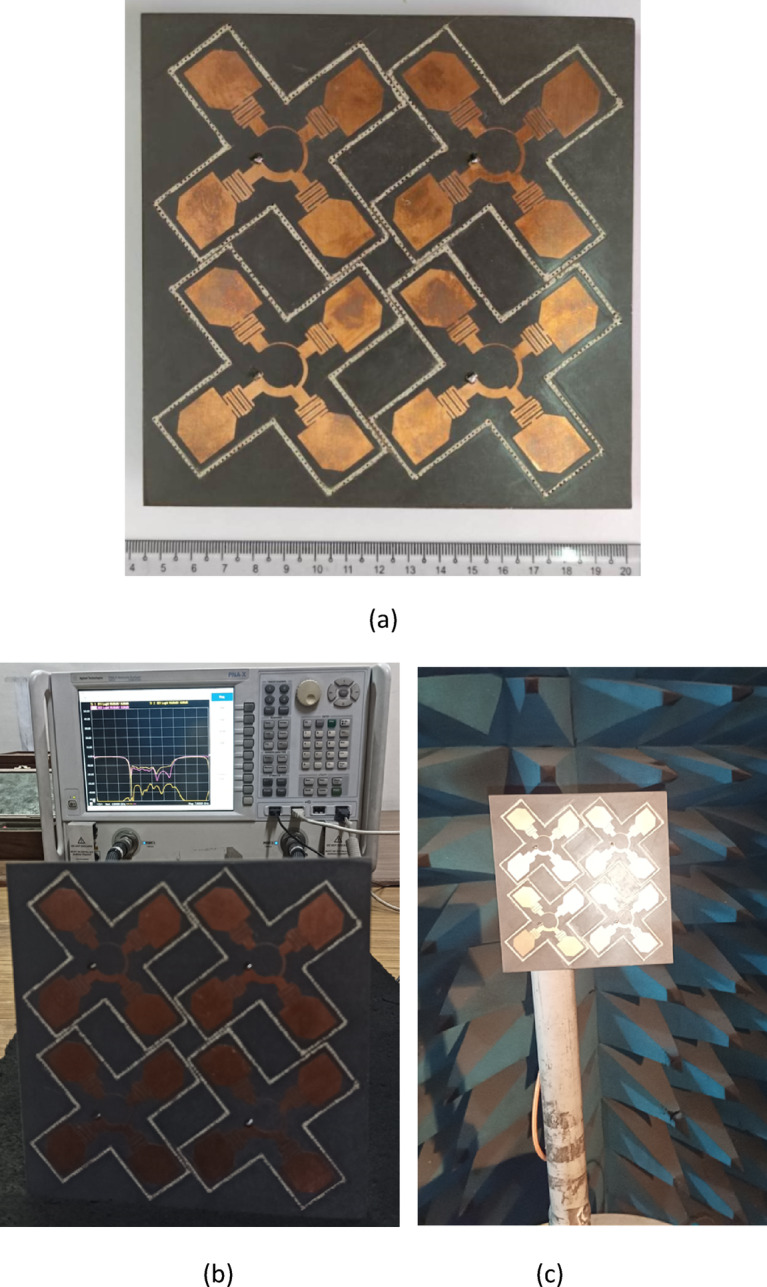
(**a**) Photograph of the fabricated prototype, (**b**) VNA measurement setup, and (**c**) AUT in a standard anechoic chamber.

The prototype of the proposed four-element co-circularly polarized MIMO filtering antenna array system is fabricated and measured to validate the proposed design concept. Figure 10a shows the photograph of the fabricated prototype. The input characteristics of the antenna system are measured using a vector network analyzer (Agilent N5247A PNA-X), and the setup for the VNA measurements is illustrated in Fig. 10b. The far-field radiation characteristics are assessed in a standard anechoic chamber as shown in Fig. 10c.

### Input characteristics

**Fig. 11:**
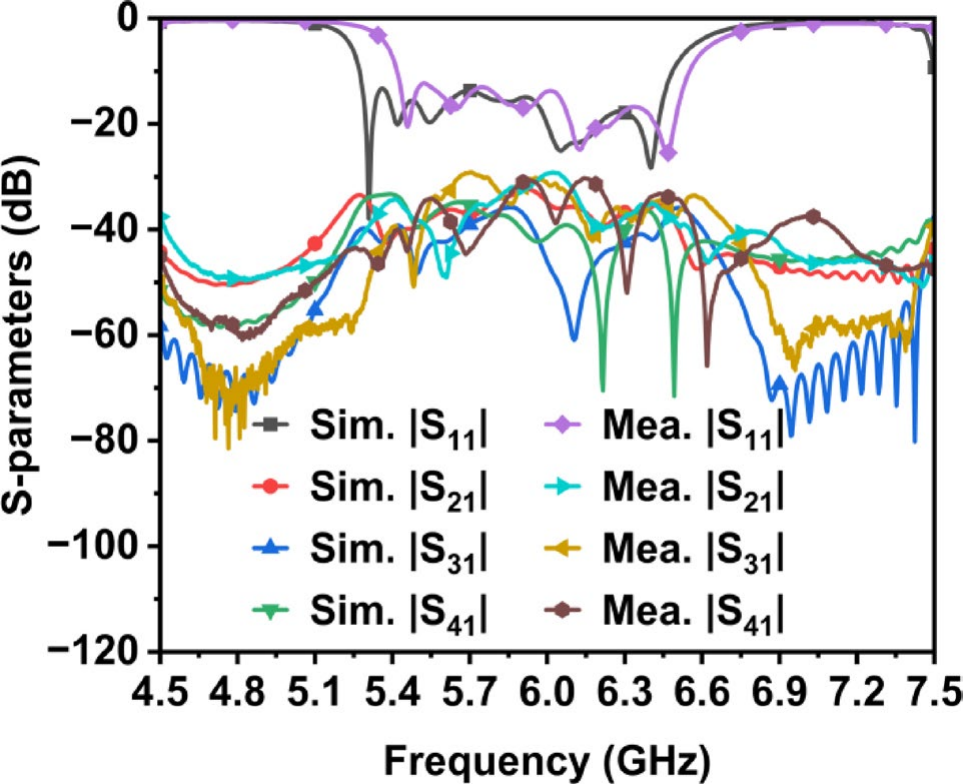
Simulated and measured S-parameters of Ant. Array 1.

The simulated and measured S-parameters for Antenna Array 1 are shown in Fig. 11. The results show a close alignment between the simulated and measured data. The measured inter-port isolation better than 31 dB is achieved throughout the entire operating bandwidth of 5.4 to 6.3 GHz.

### Far-field radiation characteristics

**Fig. 12:**
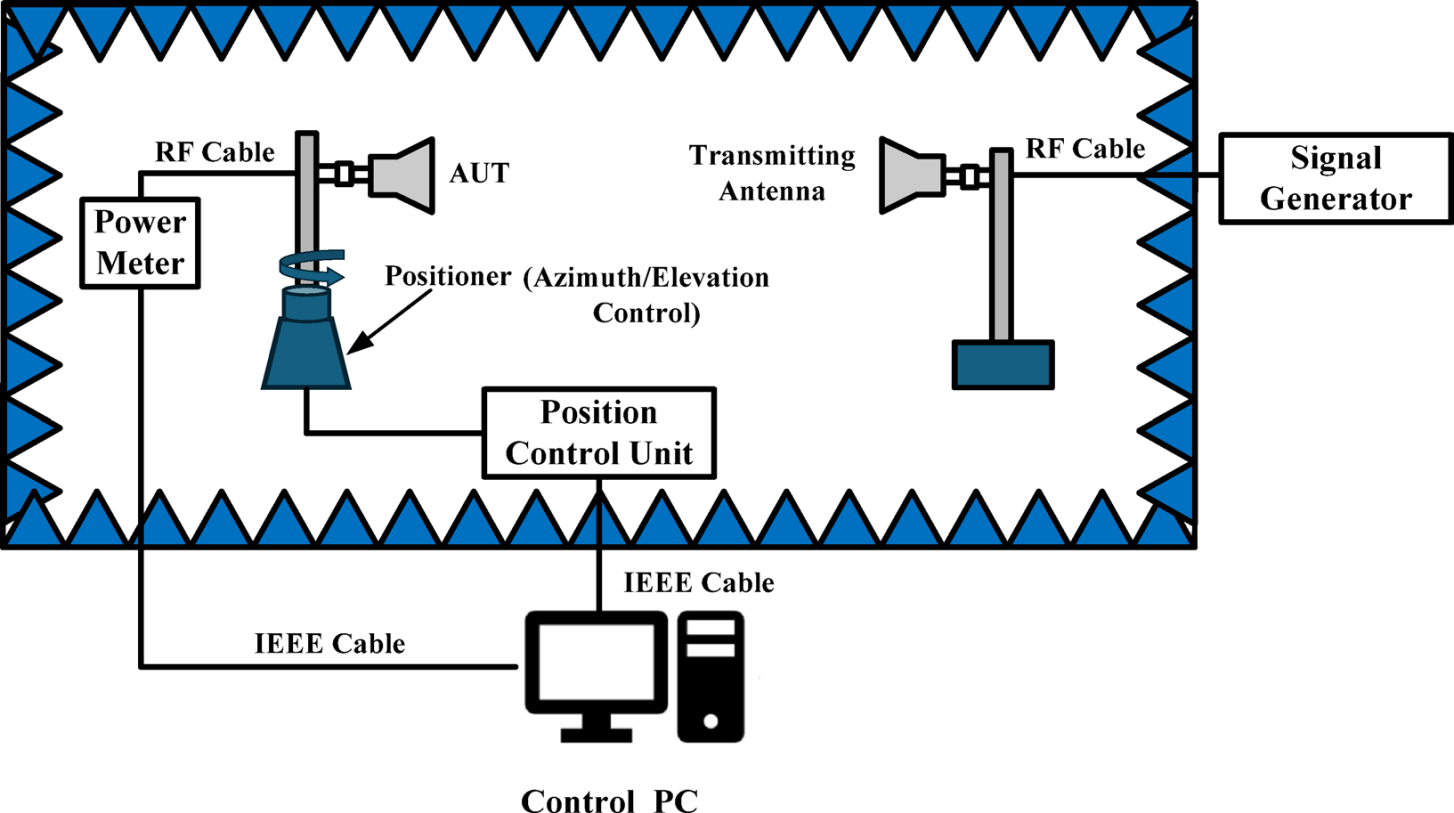
Gain measurement setup.

The realized gain of the proposed RHCP antenna is measured in a fully anechoic chamber using the Friis transmission method. The gain measurement setup is shown in Fig. 12. A calibrated standard gain horn, powered by a stable RF source, served as the transmitting antenna, while the antenna under test (AUT) was connected through low-loss cables to a power meter on the receiving side. The gain of the reference horn antenna is taken from the calibration data sheet provided by the manufacturer. The distance between the transmitting horn and the AUT is chosen according to the Fraunhofer far-field criterion. Before testing, the insertion loss of the RF cables is measured and compensated for in the gain calculation. The received power is recorded directly by the power meter, and the realized broadside gain of the AUT is calculated using the Friis equation. This involved combining the measured received power with the known gain of the horn antenna, the free-space path loss, the compensation, and the calibrated cable losses. This systematic procedure ensured an accurate and reliable estimation of the realized RHCP gain of the proposed antenna in the boresight direction.

**Fig. 13:**
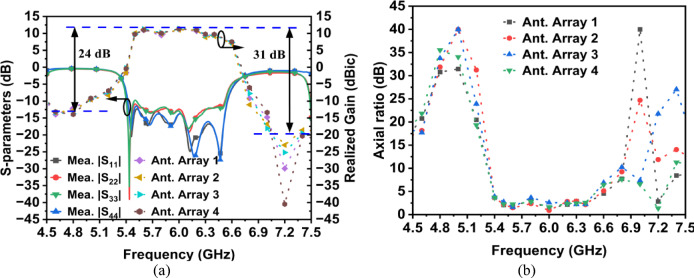
(**a**) Measured input reflection coefficient and realized gain of the antenna arrays, and (**b**) the measured axial ratio of the antenna arrays.

The measured realized gain in the boresight direction of the antenna array system is illustrated in Fig. 13a. Within the pass band of 5.4 to 6.3 GHz, the measured peak gain is 11.3 dBic, while the simulated gain is 11.74 dBic. The average measured gain across the entire operating band is approximately 10.5 dBic. The realized out-of-band suppression level measures 24 dB at the lower band (4.8 GHz) and 31 dB at the higher band (6.9 GHz), demonstrating sharp filtering characteristics. The 3-dB axial ratio measurements in Fig. 13b show that the axial ratios remain well below 3 dB throughout the entire operating band.

**Fig. 14:**
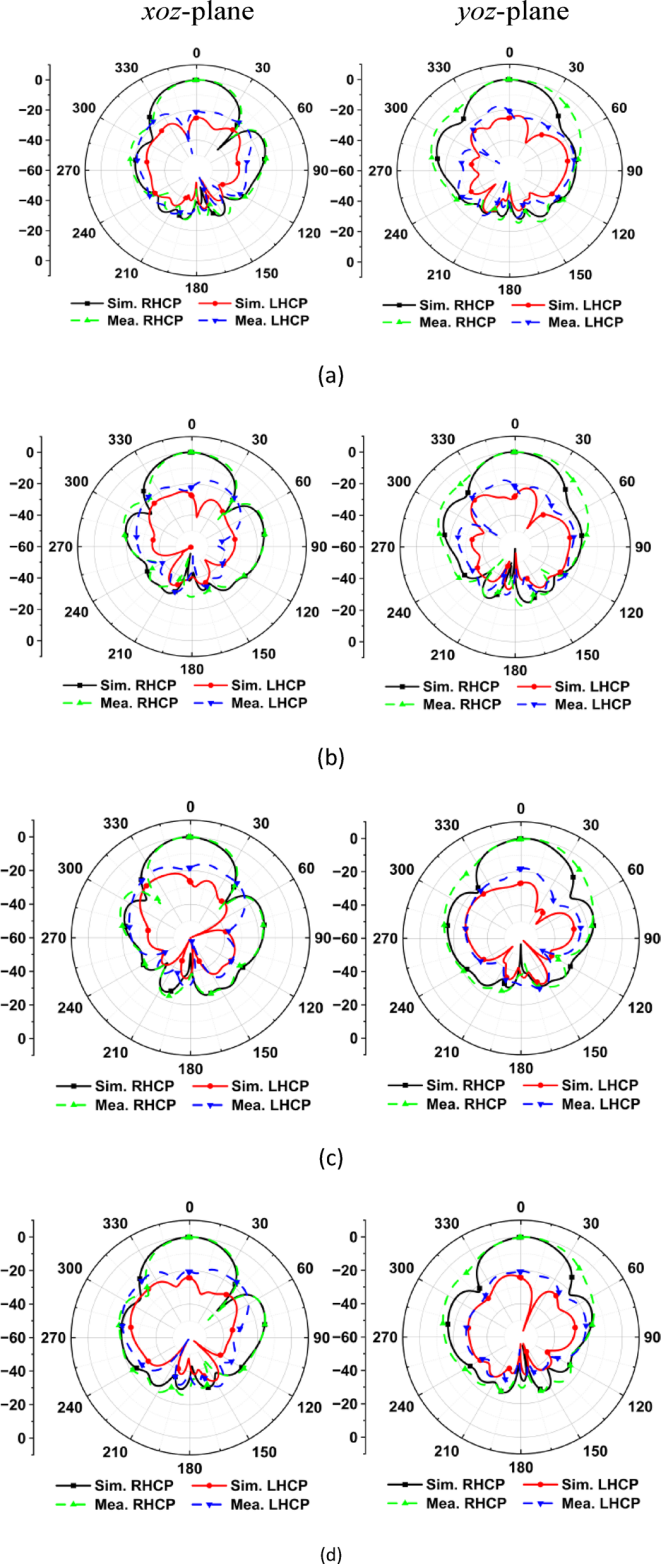
Simulated and measured radiation patterns of the proposed MIMO antenna array at 5.9 GHz for (**a**) Ant. Array 1 (**b**) Ant. Array 2 (**c**) Ant. Array 3 (**d**) Ant. Array 4.

Figure 14 illustrates the simulated and measured normalized radiation patterns of the proposed four-element CP-MIMO filtering antenna in the *xoz* and *yoz* planes. In all cases, the RHCP component forms the dominant main lobe, while the LHCP response is suppressed by more than 20 dB at boresight. This suppression confirms that each element maintains strong circular polarization purity. The measured patterns closely align with the simulated results, with the RHCP envelopes preserved. While there are some minor deviations in sidelobe magnitude and ripple, these can be attributed to fabrication tolerances and measurement chamber effects. However, these variations do not significantly impact the overall performance of the circular polarization. The RHCP beams of the four ports are consistently aligned, ensuring stable polarization and a uniform gain distribution across the array. Maintaining RHCP dominance across all elements in the MIMO configuration is particularly important in vehicular environments. In V2X channels, multipath propagation caused by surrounding vehicles, infrastructure, and road surfaces often introduces polarization distortion, with some reflected components arriving as LHCP. By ensuring that the direct-path radiation from each port is strongly RHCP-dominant, the array minimizes polarization mismatch losses and maintains high-quality connectivity. This balance of strong RHCP main lobes with suppressed LHCP components enhances link robustness and supports reliable, high-capacity MIMO communication under realistic mobile channel conditions.

The efficiency characteristics of the proposed four-port filtering MIMO antenna is thoroughly analyzed through both simulations and measurements. The radiation efficiency is measured using the Wheeler Cap method^22^. This technique eliminates mismatch effects, providing a reliable estimate of the antenna’s radiating capability. The radiation efficiency can be calculated directly from the magnitudes of the measured reflection coefficients, using Eq. (8).

8 $$\eta_{rad} = \frac{{\left| {S_{11,free} } \right|^{2} - \left| {S_{11,cap} } \right|^{2} }}{{\left| {S_{11,free} } \right|^{2} - 1}}$$

where $|S_{11,free|}$ and $|S_{11,cap} |$ denote the magnitudes of the input reflection coefficients measured in free space and under the Wheeler cap, respectively. The power efficiency, is calculated from the measured input reflection coefficient using Eq. (9).

9 $$\eta_{pwr} = 1 - \left| {S_{11} } \right|^{2}$$

**Fig. 15:**
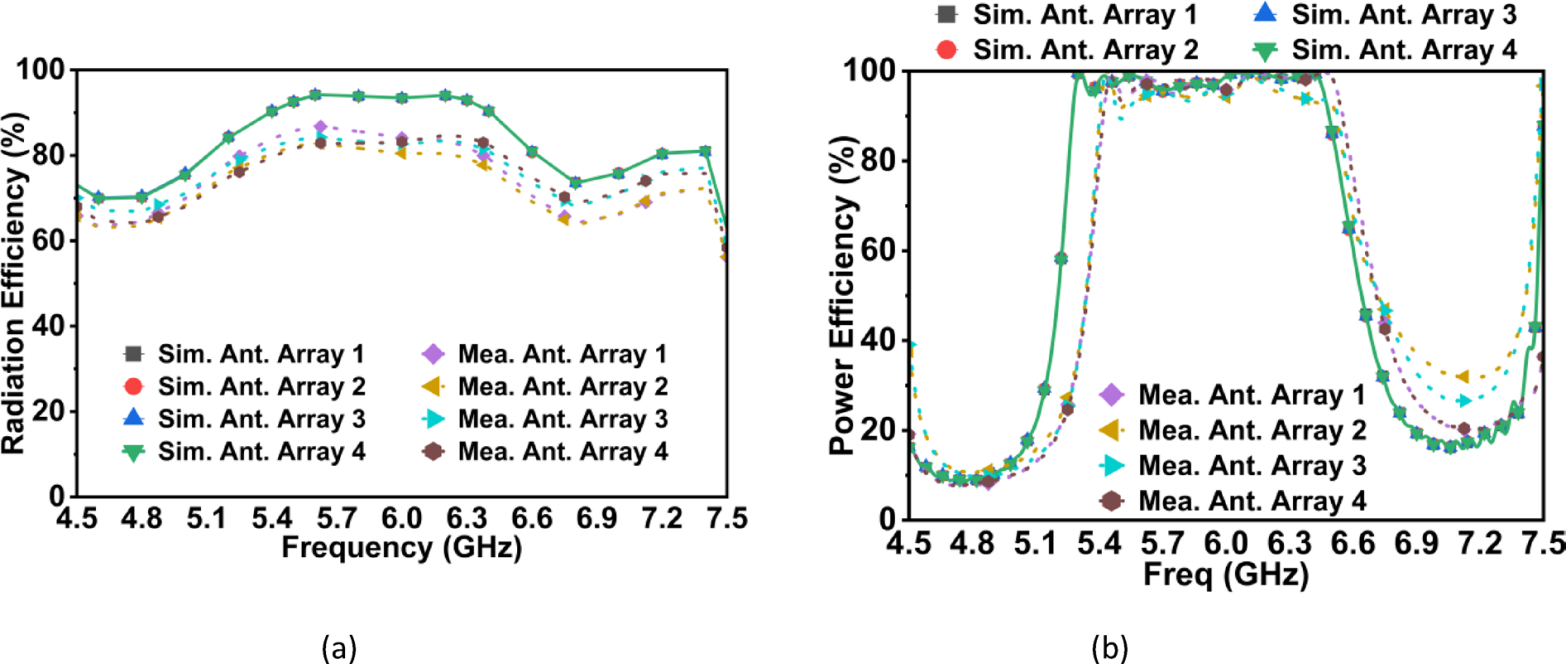
(**a**) Radiation efficiency, and (**b**) power efficiency of the proposed four-element CP-MIMO filtering antenna.

This parameter quantifies the fraction of input power that is accepted by the antenna. Figure 15 shows simulated and measured values for the radiation efficiency and power efficiency. The simulated results show that the radiation efficiency approaches 95% across the operating frequency range, indicating minimal ohmic and dielectric losses. As expected, the measured values are slightly lower than the simulations due to connector and cable effects and fabrication tolerances. However, these measured values remain consistently high, confirming that the antenna radiates efficiently even under real-world conditions. For power efficiency, both simulated and measured responses demonstrate strong acceptance of input power within the 5.4–6.3 GHz band, with minor deviations attributed to fabrication and connector effects. Outside the pass band, simulation and measurement show a natural decline in power efficiency, which is a direct consequence of the antenna’s filtering characteristics.

### MIMO performance

**Fig. 16:**
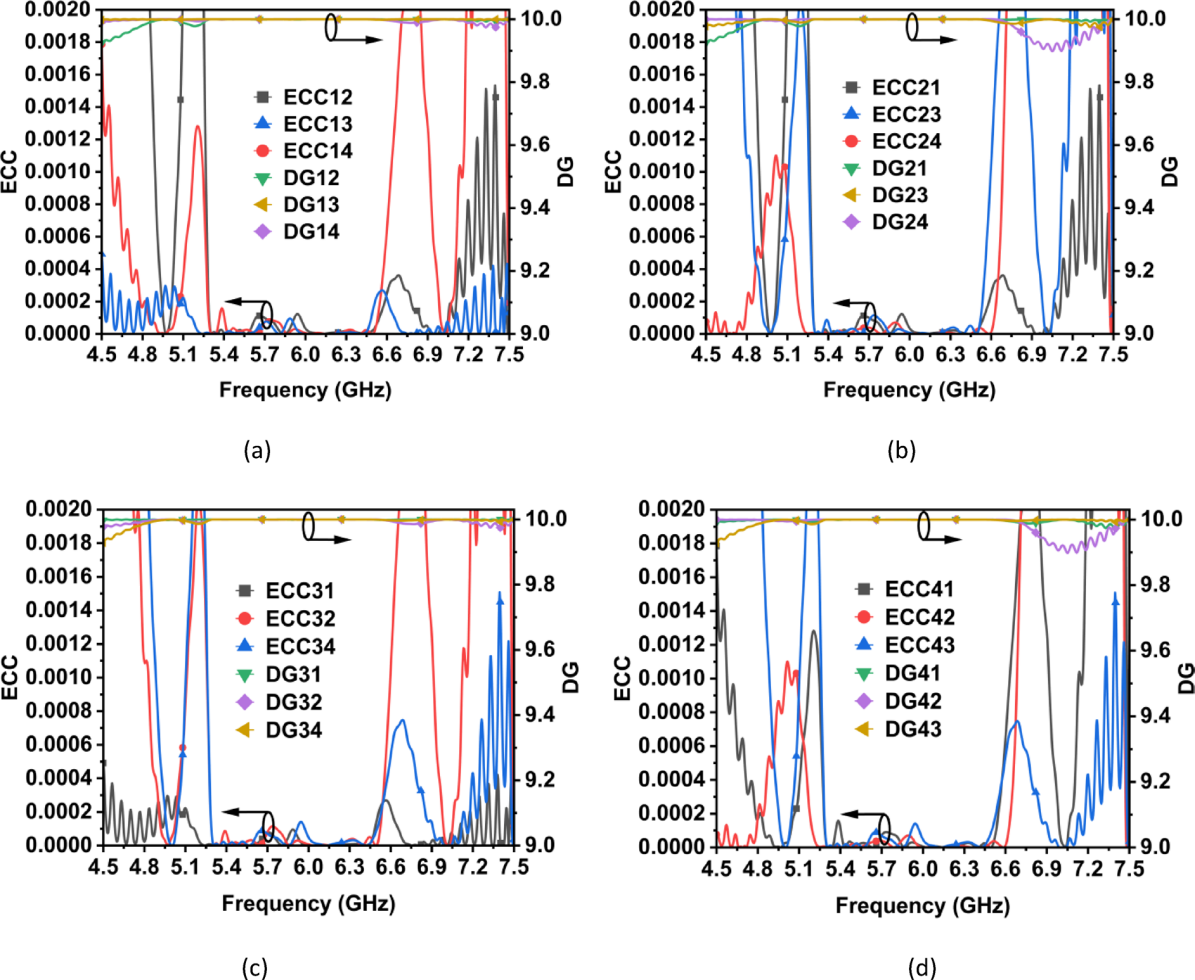
ECC and DG of (**a**) Ant. Array 1, (**b**) Ant. Array 2, (**c**) Ant. Array 3, and (**d**) Ant. Array 4.

The MIMO performance of the designed CP MIMO antenna is evaluated in terms of envelope correlation coefficient (ECC), diversity gain (DG), total active reflection coefficient (TARC), mean effective gain (MEG), channel capacity (CC) and channel capacity loss (CCL). Figure 16 shows the ECC and DG of the proposed four-element co-circularly polarized MIMO filtering antenna array system. The ECCs are evaluated from the far-field components using the method described in^23^, while DGs are evaluated from ECCs^23^. The ECC values consistently fall below 0.00013, which indicates that the MIMO antenna elements are almost uncorrelated. Additionally, the DG remains close to the desired value of 10 throughout the entire operating bandwidth.

**Fig. 17:**
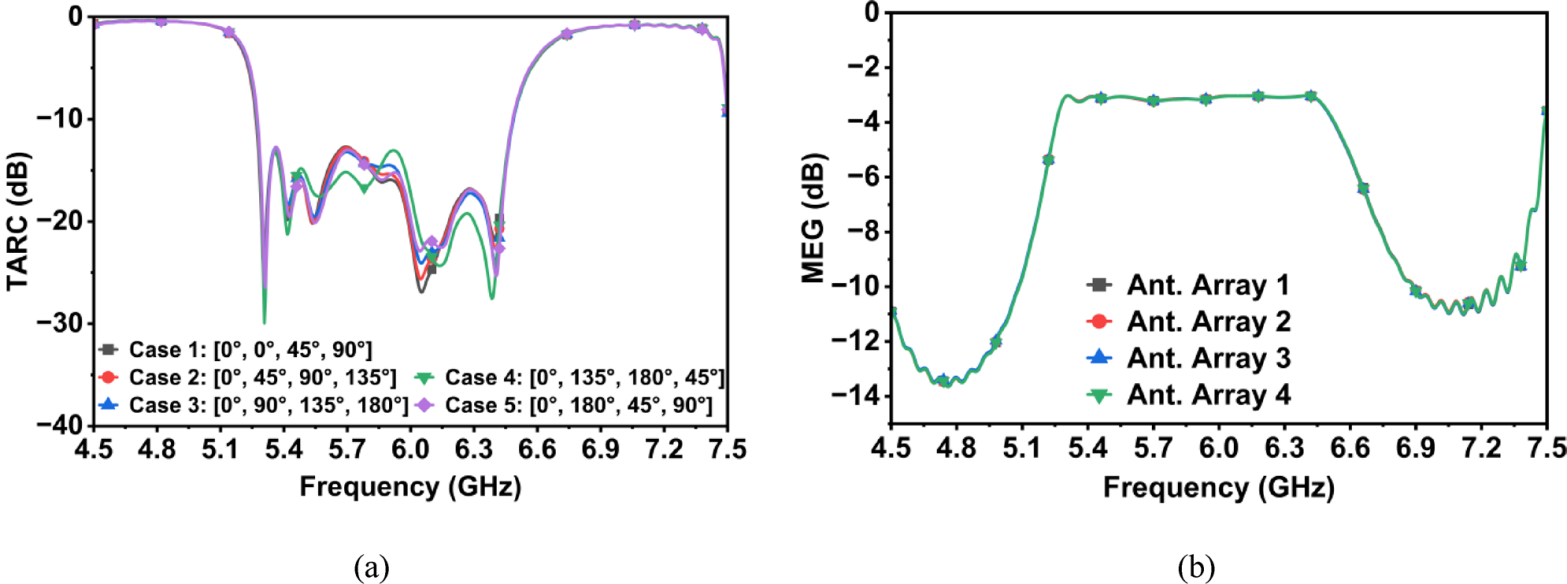
(**a**) TARC and (**b**) MEG of the proposed MIMO antenna array.

Figure 17 illustrates TARC and MEG of the designed antenna system. TARC values are evaluated over the non-uniform scenarios, where Port 1 is fixed at 0° while the other ports are varied across five different phase combinations^24^: [0°, 45°, 90°], [45°, 90°, 135°], [90°, 135°, 180°], [135°, 180°, 45°], and [180°, 45°, 90°]. The results, illustrated in Fig. 17a, indicate that TARC is mostly independent of the excitation phase, consistently remaining below the -10 dB threshold within the operating frequency range of 5.4–6.3 GHz. This behavior reflects that the antenna elements are well decoupled, allowing for reliable MIMO performance across varying excitation conditions. MEGs are calculated using the expression described in^25^ using S-parameters. As illustrated in Fig. 17b, they remain above -3.1 dB, with variation between ports less than 0.1 dB. This uniformity indicates that no element dominates or suffers in power reception, thereby confirming the array’s capability to maintain balanced performance and to support consistent diversity gain across all ports.

**Fig. 18:**
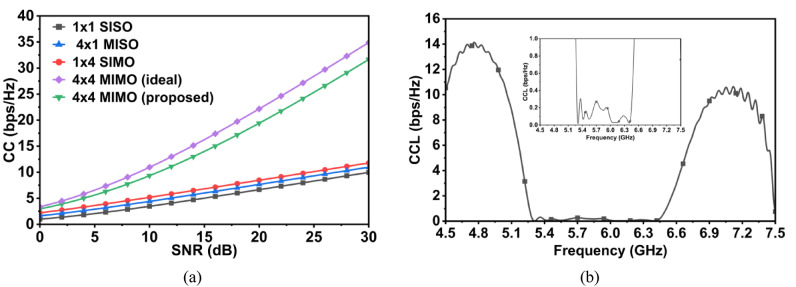
(**a**) CC and (**b**) CCL of the proposed MIMO antenna array.

Moreover, the CC and CCL of the proposed CP MIMO antenna are evaluated and the results are plotted in Fig. 18. The channel capacity as a function of SNR for single-input-single-output (SISO), multiple-input-single-output (MISO), single-input-multiple-output (SIMO), and MIMO configurations are also plotted according to the expressions in^26^. At 20 dB SNR, the SISO configuration achieves nearly 6.66 bps/Hz, representing the fundamental limit of a single transmit-receive link. The MISO configuration, shows a slight improvement by reaching ≈ 7.65 bps/Hz, reflecting the advantages of transmit diversity. However, since no additional parallel streams are created, the overall gain remains modest, and the capacity trend remains close to that of SISO. In contrast, the SIMO configuration, with one transmit and four receive antennas, shows further improvement compared to MISO, achieving about ≈ 8.46 bps/Hz at 20 dB. This enhancement results from receive diversity, as the receiver coherently combines signals from multiple antennas, leading to a higher effective SNR. A substantial gain is observed for the full 4 × 4 MIMO configuration, where the proposed antenna attains ≈ 19.40 bps/Hz. This value is very close to the theoretical benchmark of ≈ 20.15 bps/Hz for an ideal uncorrelated 4 × 4 MIMO channel, confirming that the design effectively supports both diversity and spatial multiplexing. For better understanding of the communication link, CCL is plotted in Fig. 18b. The CCL values should be below 0.5 bps/Hz for good MIMO performance^27^. For the proposed design, across the 5.4–6.3 GHz operating band, the CCL remains below 0.13 bps/Hz, indicating low port correlation and minimal impact of coupling on system capacity.

## Comparison

Table 1 provides a comparative analysis between the proposed filtering antenna unit element and other recently reported filtering patch antennas. It can be observed that the proposed unit element features a simpler single-layer design, while achieving larger ARBW and higher suppression levels compared to all the reported works. All the reported structures except^14^ and^30^ are multilayered, requiring complicated fabrication techniques and potentially leading to alignment issues. The proposed unit element achieves a peak realized gain of 11.3 dBi, which is 1.5 dB less than the value reported for the filtering patch antennas in^17^. The difference arises from the fact that the filtering patch antennas reported in^17^ utilizes SIW cavities with a relatively high profile (~ 0.134*λ*_0_), whereas the proposed unit element maintains a considerably lower profile of 0.03*λ*_0_. However, the peak realized gain can be enhanced by placing a superstrate over the proposed unit element at the expense of an increased profile. Furthermore, the proposed unit element achieves superior out-of-band suppression, surpassing that of other reported structures.

**Table 1:** Comparison of the filtering antenna unit element with the reported filtering patch antennas.

Ref./Year	*f*_0_ (GHz)	Profile (*λ*_0_)	3-dB ARBW (%)	Imp. FBW (%)	Peak gain (dBic)	No. of layers	Suppression level (dB)	Filtering method
^11^/2023	3.5	0.035	14.29	11.4	10.74	2 + air gap	21.43	Cross-coupling and open-end branch lines
^14^/2021	2.4	0.02	5.3	4.5	8.3	1	20	Parasitic elements (microstrip stubs, shorting pins and U-shaped slots)
^16^/2021	5.03	0.036	3.9	7.2	8	3	No nulls	Dual-mode SIW cavity (TE_102_/TE_201_)
^17^/2018	10.2	0.134	9.3	8.3	12.89	3	No nulls	SIW cavity filters
^28^/2020	2.4	0.019	3.8	3.8	6.1	2	18	Dispersive delay line (DDL)
^29^/2023	1.945	0.0648	8.74	15.2	7	2 + foam	22	Fusion technique: U-slot (in lower patch) + square annular slot (in upper patch)
^30^/2024	4.795	0.025	5.63	8.3	10	1	17	Embedded SIW cavity resonator coupled to circular patch
^31^/2025	4.82	0.04	8.6	11.3	6.6	2	22	Mode coupling + radiation nulls (parasitic patches, U-slot)
^32^/2025	4	0.1445	6.75	CM: 54.45 DM: 74.4	8.43	3 + air gap	20	Cross-shaped triple-stub multimode resonator
^33^/2023	2.36	0.11	9.32	17.72	7.07	2 + foam	18	Crossed slot and parasitic patches
Prop	5.85	0.03	15.4	20	11.3	1	24	Three-pole hairpin resonators-based band pass filters

*f*_0_: Centre frequency, ARBW: Axial ratio bandwidth, Imp. BW: Impedance bandwidth.

For clarity, Table 2 summarizes the comparison of the proposed CP MIMO filtering antenna with previously reported MIMO filtering antennas. As presented, the proposed CP MIMO filtering antenna employs a single-layer design, offering a wide ARBW of 15.4%, excellent inter-port isolation (> 34 dB), and the smallest minimum edge-to-edge separation among the reported designs. Moreover, the proposed CP filtering MIMO antenna exhibits higher suppression level. Owing to its outstanding out-of-band suppression, excellent inter-port isolation, wide ARBW, superior radiation characteristics, and exceptional MIMO performance, the proposed CP MIMO filtering antenna stands out as a strong candidate for emerging vehicular communication technologies and standards. Based on the above discussion, the salient key features of the proposed CP filtering MIMO antenna can be summarized as follows:

- A compact, single-layer modified sequential-phase feed network with integrated third-order hairpin band pass filters is developed, which directly excites four RHCP patches with progressive 0°/90°/180°/270° phases, simultaneously achieving high band pass selectivity and wide ARBW.
- For the first time, a uniform 120° geometric rotation of all elements in a compact four-port circularly polarized MIMO array is demonstrated to preserve global RHCP radiation characteristics while orthogonally orienting adjacent patches, thereby inherently suppressing mutual coupling and ensuring high inter-port isolation.
- To the best of our knowledge, this is the first instance where via-strip caging is reported to enclose the entire circularly polarized MIMO element that enhances inter-port isolation, thereby enabling scalable MIMO array expansion while preserving co-polar RHCP radiation characteristics.
- The proposed MIMO filtering antenna achieves a 15.4% ARBW, 24 dB out-of-band suppression, peak realized gains of 11.3 dBic, and inter-port isolations better than 34 dB, all realized on a single layer with a compact edge-to-edge spacing of only ~ 0.012* λ*_0_.

**Table 2:** Comparison of the proposed filtering MIMO antenna with reported works.

Ref./Year	Antenna Type	*f*_0_ (GHz)	E-E gap (*λ*_0_)	Profile (*λ*_0_)	No. of layers	3-dB ARBW (%)	Imp. BW (%)	Peak gain (dBic/dBi)	Min. isola-tion (dB)	No. of ports	Filter-ing	ECC/CCL (bps/Hz)	Suppression level (dB)
^34^/2023	DRA	4.215	0.54	0.2166	1	9.29	9	9.2	25	2	Yes	< 0.002/0.2	7
^35^/2024	Patch	3.5	NA	0.144	2 + air gap	13.1	21.8	12.3	15	2	Yes	< 0.02/NA	14
^36^/2023	DRA	4.15	NA	0.0138	1	No	37.5	4	14	4	Yes	< 0.002/NA	4
^37^/2022	Patch	3.35	0.72	0.012	1	No	33.5	NA	16	4	Yes	< 0.03/NA	NA
^38^/2025	Patch	2.535	0.02	0.013	1	No	14.6	0.6	19.8	4	Yes	NA/NA	14.2
**Prop**	Patch	**5.85**	**0.012**	**0.03**	1	**15.4**	**20**	**11.3**	**34**	4	Yes	< 0.00013/ 0.13	24

E-E gap: Edge-to-edge gap, *f*_0_: Centre frequency, ARBW: Axial ratio bandwidth, Imp. BW: Impedance bandwidth, NA: Not available.

Significant values are in [bold].

In future work, attention could be given to filtering and polarization reconfigurability as well as advanced MIMO configurations^39,40^. Filtering reconfigurability can be realized by incorporating diodes for continuous frequency tuning or RF switches for choosing discrete bands within the feed network. For polarization reconfigurability, corner-truncated patches can be employed, enabling one diagonal to generate RHCP and the other LHCP^41,42^.

## Conclusion

This communication presents a compact, single-layer, four-element MIMO filtering antenna array system designed for vehicular communications operating in the range of 5.4–6.3 GHz. A novel sequential phase feed network that substitutes *λ*/4 transformers with three-pole hairpin resonators adds the filtering capabilities to a 2 × 2 arrangements of sequentially rotated truncated corner rectangular patch antennas. The MIMO filtering antenna array elements achieve notable out-of-band frequency suppression, surpassing 24 dB in the lower stop band and 36 dB in the upper stop band, indicating a sharp filtering response. Furthermore, these elements demonstrate excellent inter-port isolation, exceeding 34 dB throughout the operating bandwidth of 5.4–6.3 GHz. The high isolation level is accomplished by rotating the antenna arrays along the central axis by 120° and creating a cage to confine the electromagnetic fields. The proposed MIMO filtering antenna array system holds significant potential for supporting emerging technologies and standards in vehicular communication. Future work will focus on filtering reconfigurability, polarization reconfigurability and higher order MIMO configurations. Filtering reconfigurability can be achieved by integrating varactor diodes for continuous tuning or PIN/RF-MEMS switches for discrete band selection within the feed network. As these modifications are confined to the feed, the radiator geometry, ARBW, and polarization purity remain unaffected. Polarization reconfigurability can be realized in corner-truncated patches, where one diagonal yields RHCP and the other LHCP. By employing PIN diodes, varactors, or RF-MEMS switches to activate auxiliary patches at opposite corners, the antenna can switch between the two polarization states while maintaining its filtering response, axial-ratio bandwidth, and radiation efficiency. The geometric rotation principle and via–strip electromagnetic cage can achieve high isolation in dense arrays, supporting large-scale and massive-MIMO systems with reduced surface- and space-wave coupling.

## Data Availability

The data used to support the findings of this study are included in the article.
